# Non-Alcoholic Components in Huangjiu as Potential Factors Regulating the Intestinal Barrier and Gut Microbiota in Mouse Model of Alcoholic Liver Injury

**DOI:** 10.3390/foods11111537

**Published:** 2022-05-24

**Authors:** Yi Yang, Zhilei Zhou, Yufei Liu, Xibiao Xu, Yuezheng Xu, Weibiao Zhou, Shuguang Chen, Jian Mao

**Affiliations:** 1 National Engineering Research Center of Cereal Fermentation and Food Biomanufacturing, School of Food Science and Technology, Jiangnan University, Wuxi 214122, China; Yy_sherlock@163.com (Y.Y.); zhouzl1985@126.com (Z.Z.); lyfsee1993@163.com (Y.L.); 2 Shaoxing Key Laboratory of Traditional Fermentation Food and Human Health, Jiangnan University (Shaoxing) Industrial Technology Research Institute, Shaoxing 312000, China; 15305693916@163.com (Y.X.); weibiao@nus.edu.sg (W.Z.); 3 Jiangsu Provincial Engineering Research Center for Bioactive Product Processing, Jiangnan University, Wuxi 214122, China; 4 National Engineering Research Center for Huangjiu, Zhejiang Guyuelongshan Shaoxing Wine Co., Ltd., Shaoxing 312000, China; wsht520520@163.com; 5 Department of Food Science and Technology, National University of Singapore, Science Drive 2, Singapore 117542, Singapore; 6 Department of General Surgery, Peking Union Medical College Hospital, Chinese Academy of Medical Sciences and Peking Union Medical College (CAMS & PUMC), Beijing 100730, China

**Keywords:** hepatic injury, Huangjiu, gut microbiome, intestinal barrier function, short-chain fatty acids

## Abstract

Different alcoholic beverages and drinking patterns might exert divergent impacts on alcoholic liver disease (ALD) progression. Whether the abundant non-alcoholic components (NAC) in fermented wine could alleviate ethanol (EtOH)-induced adverse influences on the liver remains unknown. Hence, the chronic ALD mouse model was established to compare the effects of Huangjiu (a typical fermented wine) and EtOH feeding on the liver, intestinal barrier, gut microbiota, and intestinal short-chain fatty acids (SCFAs) content. Although Huangjiu intake led to slight hepatic steatosis, it mitigated oxidative stress, inflammation, and intestinal damage relative to EtOH intake. In comparison with EtOH feeding, Huangjiu significantly improved the intestinal barrier integrity and reduced hepatic lipopolysaccharide levels by up-regulating the expression of intestinal tight junction proteins (ZO-1 and occludin) and antimicrobial activity peptides (Reg3β and Reg3γ). The administration of Huangjiu NAC partially restored alcohol-induced gut microbiota dysbiosis via recovering the abundance of *Lactobacillus*, *Faecalibaculum*, and *Akkermansia*. Moreover, mice receiving Huangjiu showed higher SCFAs levels (such as acetic acid and butyric acid) than those receiving EtOH. Huangjiu consumption resulted in lower hepatotoxicity than pure EtOH, at the same alcohol dose. The NAC in Huangjiu might attenuate the progression of ALD by regulating intestinal barrier function and microbiota-meditated gut ecology.

## 1. Introduction

Alcohol consumption is correlated with a series of diseases, such as liver injury, hypertension, stroke, and cancer [1]. Following the latest report, about 2.3 billion people consumed an average of 32.8 g of pure EtOH daily worldwide in 2018, and these data increased consecutively [2]. The liver is the primary site of alcohol metabolism and alcoholic liver disease (ALD) has been one of the leading causes of worldwide liver cirrhosis and liver-related death. Furthermore, ALD might induce other clinical disorders, such as atherosclerosis, hyperlipidemia, and hypertension [3].

Reactive oxygen species (ROS) generated during EtOH metabolism could modify and inactivate cellular macromolecules, such as protein, lipid, and nucleic acid, destroying cells’ normal structure and function [4]. Furthermore, ROS might cause lipid peroxidation and promote hepatic oxidative stress throughout consuming intracellular glutathione (GSH) and antioxidant enzymes, thus, promoting ALD progression [5]. Additionally, malonyldialdehyde (MDA), a lipid peroxidation product, may also stimulate the immune cell response and induce the expression of inflammatory cytokines, which further contribute to liver inflammation [6].

Accumulating evidence illustrated that gut microbiota and its metabolites, the intestinal barrier, and the gut–liver axis played a crucial role in ALD progression [6,7]. Long-term excessive EtOH intake could induce decreased diversity and dysbiosis of gut microbiota and destroy the intestinal mucosa and tight junction (TJ) proteins [6,8,9]. The elevated levels of bacterial endotoxin (lipopolysaccharides, LPS) and the enhanced intestinal permeability to LPS following chronic alcoholism lead to the transfer of excess endotoxin from the intestine to the portal vein and then into the liver and blood circulation [10]. LPS was combined with the specific receptor CD14 and Toll-like receptor 4 (TLR4) of hepatic Kupffer cells to activate the nuclear factor κB (NF-κB) and then released numerous cytokines and inflammatory mediators, including tumor necrosis factor-α (TNF-α), interleukin-1β (IL-1β), and interleukin-6 (IL-6), thereby resulting in hepatocyte steatosis, inflammation, and hepatocyte necrosis [11]. Short-chain fatty acids (SCFAs), derived from metabolites of intestinal microbiota, have recently been proved to be essential for alleviating inflammation in different chronic metabolic diseases, and might contribute to the attenuation of ALD by reducing the inflammatory response [12,13,14].

Chronic alcohol abuse is considered the principal reason for ALD-related morbidity and mortality [15]. However, alcohol consumption has been around for thousands of years as a prevalent traditional custom in many countries. Recently, the relationships among alcoholic beverage types, drinking patterns and health, the functional characteristics of non-alcoholic components (NAC) in alcoholic beverages, the interactions between NAC and the alcohol in alcoholic drinks, and their impacts on human health have aroused widespread concern. Epidemiological studies have indicated that there could be a good association between mild to moderate alcohol consumption and health [16,17,18]. Several phenolic compounds in red wine confer strong protection against metabolic, cardiovascular, and neurodegenerative diseases [19,20]. Therein, resveratrol and ellagic acid have been reported to exhibit anticancer activities by inhibiting the cell cycle, inhibiting tumor cell proliferation, inducing cell apoptosis, improving inflammation, and arresting angiogenesis [20,21]. The iso-α-acids and xanthohumol in beer were found to have a protecting role in some neurodegenerative diseases [22]. Additionally, the total polar lipid extracts of beer exhibited antithrombotic properties of inhibiting platelet-activating factor and thrombin-induced human platelet aggregation [23]. Sake concentrate and its unique sugar component ethylα-D-glucoside were reported to inhibit galactosamine-induced liver injury by suppressing interleukin 6 (IL-6) production and liver DNA fragmentation [24].

As a typical representative of traditional fermented wine, Huangjiu (Chinese rice wine) has more than 5000 years of brewing history. It is primarily brewed with glutinous rice as raw material, wheat Qu, and yeast (such as *Saccharomyces cerevisiae*) as saccharifying agents and starter. Wheat Qu contained complex microflora, comprising *Aspergillus* spp., *Rhizopus* spp., *Fusarium*, *Mucor*, *Saccharopolyspora*, lactic acid bacteria, and yeasts secrete a diversity of enzymes, primarily liquefying enzyme, saccharifying enzyme, proteolytic enzyme, peptidase, lipase, and esterase [25]. During the process of wheat Qu production, pre-fermentation, and post-fermentation, numerous metabolic enzymes formed by microorganisms promote the transformation of high molecular substances (for example, starch and proteins) in glutinous rice and wheat into polysaccharides, oligosaccharides, ethanol, peptides, and amino acids, and result in the generation of polyphenol, organic acids, and flavor substances [26,27,28,29]. Previous studies revealed that Huangjiu polysaccharides had antioxidant and anti-tumor activities and could effectively improve cytokine levels and immunoglobulins and complements related to immunomodulatory activity in the serum of immunodeficient mice [28,30]. Furthermore, more than 500 types of Huangjiu peptides were identified, some of which were stated to have prominent antioxidant, immunoregulatory, cholesterol-lowering, ACE inhibitory, and gut-microbiota-regulating activities [25,31,32,33]. Moreover, it was demonstrated that peptides and polyphenols of Huangjiu had beneficial effects on reducing atherosclerosis and down-regulating serum lipid levels [23]. However, scarce evidence is available about the possible protective effects and mechanisms of bioactive ingredients in Huangjiu on alcohol-induced liver injury. Therefore, the classic Lieber-DeCarli model of chronic alcoholic liver injury was implemented to imitate the human long-term drinking pattern and evaluate the partial antagonistic effects of NAC in Huangjiu on alcohol-induced hepatotoxicity. Additionally, the intestinal barrier function and community structures of gut microbiota were investigated to explore the potential protective mechanisms of Huangjiu NAC against ALD. The current study provided a theoretical basis for further targeting the core substances with liver protection and alleviating the EtOH-induced health impairment.

## 2. Materials and Methods

### 2.1. Characterization of the Main Components in Three Types of Huangjiu

Considering that semi-dry Huangjiu is the mainstream of market consumption, three semi-dry Huangjiu (type A, type B, type C) aged for 5 years from three representative manufacturers were screened for a comparative study on hepatotoxicity with pure EtOH. The compositions of Huangjiu including ethanol, total protein, total phenolics, free amino acids, total sugars, oligosaccharides, polysaccharides and peptides <3 kDa, were determined as described in Section 1, Supplementary Materials. The concentration details of the eight fundamental components in Huangjiu are shown in Table 1.

### 2.2. Animal and Experimental Design

**Figure 1:**
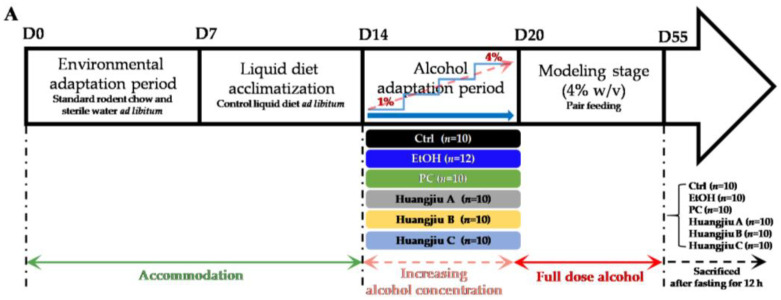

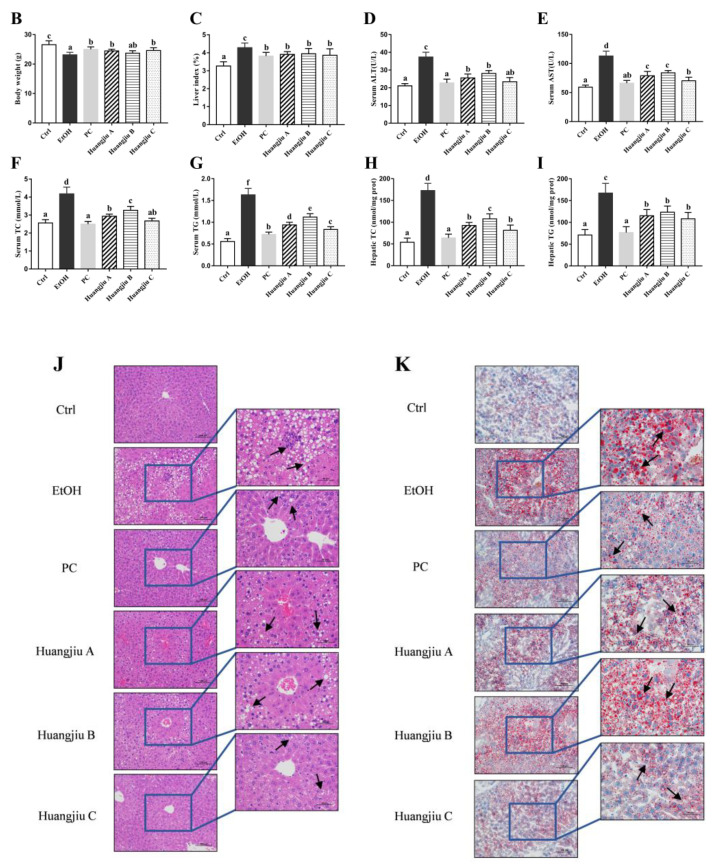

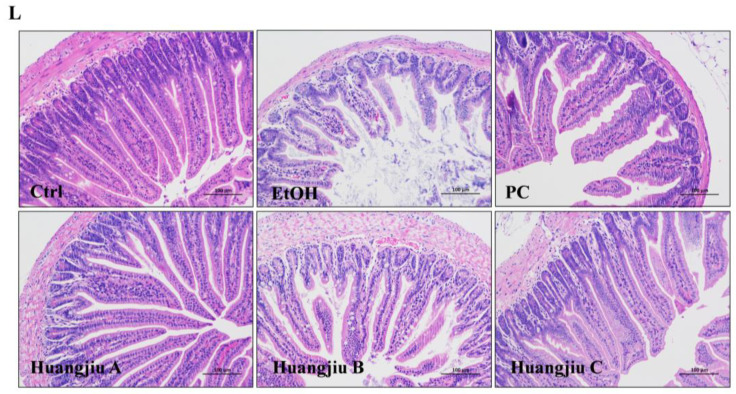
Effects of Huangjiu interventions on murine phenotype, biochemical and pathological indicators related to ALD. (**A**) Animal feeding schedule for Lieber-DeCarli model of chronic alcoholic liver injury. This animal model included the pair-fed control group (Ctrl, *n* = 10), the ethanol group (EtOH, *n* = 12), the positive control group (PC, *n* = 10), and the Huangjiu groups (*n* = 30; 10 replicates for each type of Huangjiu). Mice were progressively adapted to the control liquid diet, then an ethanol liquid diet (1–4%, *w/v*), and finally fed a 4% *w/v* ethanol liquid diet for 5 weeks. (**B**) Body weight. (**C**) Liver index. The liver index (%) was calculated as grams per 100 g body weight. (**D**) Levels of serum ALT. (**E**) Levels of serum AST. (**F**) Levels of serum TC. (**G**) Levels of serum TG. (**H**) Levels of hepatic TC. (**I**) Levels of hepatic TG. (**J**,**K**) Histopathological analysis of murine liver (by (**H**,**E**) and Oil Red O staining). Magnification ×200 and ×400. Thin arrows indicated lipid droplets. (**L**) Histological analysis of the murine ileum. Magnification ×200. Bar plot shown as mean ± standard deviation (SD) (*n* = 10). Different lowercase letters indicated statistically significant differences (*p* < 0.05) between the groups using one-way ANOVA followed by Fisher’s LSD test. ALT, alanine aminotransferase; AST, aspartate aminotransferase; TC, total cholesterol; TG, total triglyceride; H&E, hematoxylin and eosin.

Male pathogen-free (SPF) C57BL/6J mice (8 weeks old, weighing 22 ± 2 g) were obtained from GemPharmatech Co., Ltd. (Jiangsu, China) Laboratory Animal Services Center (SPF, SCXK (Jiangsu) 2019-0009). The mice were housed two or three per cage throughout the experiments in a strictly controlled environment with a 12 h light–dark cycle. The experiment was carried out in accordance with the guidelines of Jiangnan University’s Ethics Committee (SPF, SYXK (Jiangsu) 2016-0045) (Approval No. JN. No20201130c1400324[349]). For the first week, standard rodent chow and sterile water were provided ad libitum. After a week of acclimating to the control liquid diet, all mice were randomly assigned to one of six groups, namely the control group (Ctrl), the ethanol group (EtOH), the positive control group (PC, silymarin, 86 mg/kg/day), the Huangjiu A group, the Huangjiu B group, and the Huangjiu C group. Prior to modeling, the five alcohol-fed groups were given a six-day alcohol adaptation period during which the alcohol concentration in the liquid diet was gradually increased from 1% to 4% (*w/v*) [13]. The Lieber-DeCarli liquid diet containing alcohol (4% *w/v*; EtOH, PC, Huangjiu A, Huangjiu B, and Huangjiu C) or isocaloric maltose dextrin (Ctrl) was isocalorically pair-fed to all groups of mice (TROPHIC Animal Feed High-tech Co., Ltd. Jiangsu, China) [13]. In addition, three Huangjiu groups’ modified alcohol liquid diets were purchased from the high-tech platform of experimental animal feeds (TROPHIC Animal Feed High-tech Co., Ltd. Jiangsu, China). In brief, Huangjiu completely provided EtOH (4% *w/v*) in the liquid diet formula of Huangjiu groups. The inherent carbohydrate, protein and fat in Huangjiu had been accurately calculated based on the Lieber-DeCarli formulation to partially replace the carbohydrate, protein and fat involved in the liquid diet formula with equal calories. The regular-type Lieber-DeCarli liquid diet formula is presented in Table S1, Supplementary Materials. Silymarin was reported to exert protective effects against a variety of liver injuries and was widely used as a positive control in the ALD model [13]. The silymarin dose used in this study (Sigma Chemical Co., St. Louis, MO, USA) is based on the clinical dose of silymarin capsule [34]. After five weeks of modeling, each mouse was placed in its own sterile room to collect feces, which were then snap-frozen with liquid nitrogen. After a 12 h fast, the mice were euthanized. The serum was carefully collected and stored at −80 °C right away. The liver tissues were collected in their entirety, washed, weighed and cut into portions. Following that, a portion of the liver tissues was fixed in a 4% paraformaldehyde solution, while another portion was directly embedded in an optimal cutting temperature (OCT) compound (Sakura, Torrance, CA, USA) for histopathological analysis. The remaining liver tissues were kept at −80 °C. Ileum tissues were rinsed and dried in cold PBS. For sectioning, a portion of the ileum was fixed in a 4% paraformaldehyde solution, and the remainder of the intestines were stored at −80 °C. A basic overview of the experimental design for chronic ALD mouse model is outlined in Figure 1A. The data of 10 mice in each group were randomly collected for the experimental analysis.

### 2.3. Histopathological and Immunofluorescence Assessment

After being fixed for 24 h, the liver and ileum tissues were embedded in paraffin, cut into 4 μm thick sections and stained with hematoxylin and eosin (H&E) to evaluate the histological characteristics. For lipid accumulation determination, the frozen liver samples embedded in OCT compound were sliced and stained with Oil Red O solution. The above stained tissue sections were observed with Nikon Eclipse E100 (Nikon Corporation, Chiyoda Ward, Tokyo, Japan). For immunofluorescence assessment, the frozen ileum slices were incubated with anti-ZO-1 and anti-occludin antibodies (1:500; Servicebio, Wuhan, China) at 4 °C overnight, followed by incubation with an FITC-conjugated secondary antibody (Alexa fluor 488 goat anti-rabbit, 1:400; Servicebio, Wuhan, China) at 37 °C in dark for 50 min. Finally, the images were observed by a Nikon Eclipse C1 fluorescence microscope (Nikon Corporation, Chiyoda Ward, Tokyo, Japan) and recorded by a Nikon DS-U3 imaging system (Nikon Corporation, Chiyoda Ward, Tokyo, Japan). The positive area density of immunofluorescence was determined using Aipathwell image analysis software (Servicebio, Wuhan, China) and expressed as the ratio of positive cumulative optical density IOD value to the tissue area, which reflected the average depth of positive signal in the whole measurement area.

### 2.4. Biochemical Analysis

The alanine aminotransferase (ALT), aspartate aminotransferase (AST), total cholesterol (TC) and total triglyceride (TG) levels in serum were measured with commercial Liquid Sample Enzymatic Assay Kits (Applygen Technologies Inc., Beijing, China). The total TC and TG contents in liver were determined using the High-Fat Sample Cholesterol and Triglyceride Enzymatic Assay Kits (for Liver and Adipocytes) (Applygen Technologies Inc., Beijing, China) following the manufacturer’s protocols. Protein concentration was detected using the BCA Protein Concentration Assay Kit (Beyotime Biotechnology, Shanghai, China). Hepatic TC and TG levels were normalized to hepatic total protein and expressed as nmol/mg of prot. “Prot” is the abbreviation of “protein”.

### 2.5. Estimation of GSH Levels, Antioxidant Enzyme Activities and Lipid Peroxidation

The 10% (*w/v*) liver homogenates were prepared based on a previous report [4]. Briefly, the frozen liver tissue was weighed and homogenized in an ice-cold PBS (pH7.4; 1:9, *w/v*). After centrifugation at 12,000× *g* for 30 min at 4 °C, the supernatant was obtained to measure GSH and MDA levels, as well as glutathione peroxidase (GPx), superoxide dismutase (SOD) and catalase (CAT) activities using commercial assay kits (Beyotime Biotechnology, Shanghai, China) according to the protocols of corresponding manufacturer. The results were normalized to hepatic total protein.

### 2.6. Hepatic LPS Content Determination

The hepatic LPS levels in mice were determined by the commercial ELISA kit (Cusabio, Wuhan, China) according to the instructions of the manufacturer.

### 2.7. Pro-Inflammatory Cytokine Level Measurement

The preparation procedure of 10% (*w/v*) liver homogenates is consistent with that described in 2.5. Supernatants were collected for the examination of hepatic levels of pro-inflammatory cytokines, including TNF-α, IL-6 and IL-1β, with Mouse Uncoated ELISA Standard kits (Thermo Fisher Scientific Inc., Sunnyvale, CA, USA), following the instructions of the manufacturer. The results were normalized to hepatic total protein and described as pg/mg of prot.

### 2.8. Quantitative RT-PCR Analysis

Total RNA from ileum tissue was extracted using Trizol reagent (Servicebio, Wuhan, China), and then reverse transcribed with a PrimeScript RT Master Mix Kit (TaKaRa Bioengineering Inc., Beijing, China) according to a standard technique to synthesize cDNA. The quantitative analysis of real-time PCR was conducted in triplicate and carried out on an ABI Q6 Real-Time PCR system (Applied Biosystems, Inc., Carlsbad, CA, USA) using the SYBR Green qPCR Master Mix (High ROX) (Servicebio, Wuhan, China). The primer sequences of GAPDH, ZO-1, Occludin, Reg3β and Reg3γ are listed in Table 2. The results were normalized to GAPDH expression and calculated using the 2^−^^△^^△Ct^ method.

### 2.9. Gut Microbiota Assessment

Total microbial DNA was extracted from mouse feces using the Fast DNA Spin Kit for Feces (MP Biomedicals, Santa Ana, CA, USA) following the manufacturer’s instructions. Successful DNA isolation was examined by agarose gel electrophoresis. PCR amplification was carried out by using the hypervariable regions (V3–V4) of bacterial 16S rDNA. The purified amplicons were pooled in equimolar ratios and sequenced on an Illumina Miseq platform (Illumina Inc., San Diego, CA, USA). The sequenced data were analyzed using QIIME toolkit and R packages [13].

### 2.10. Quantitative Analysis of SCFAs

The fecal SCFAs were isolated according to a previously described method with some modifications. Briefly, the fecal samples weighing 50 mg were homogenized in 500 μL of saturated NaCl solution at 70 Hz for 2 min. After 40 μL of 9% H_2_SO_4_ (*v/v*) was added, the mixtures were shaken for 30 s and mixed well. Then, 800 μL of anhydrous ether was added to extract the SCFAs and the mixtures were uniformly oscillated for 30 s. Subsequently, the mixtures were centrifuged at 12,000× *g* for 15 min (4 °C) and the collected supernatants were mixed with 0.25 g of anhydrous Na_2_SO_4_ to remove the water. After being centrifuged at 12,000× *g* for 15 min (4 °C), the supernatants with SCFAs were transferred into the vials and finally measured by gas chromatography-mass spectrometer (GC-MS). The separation was carried out on an Rtx-Wax column (Guangzhou Ai Xin biology science and technology co., ltd., Guangzhou, China) with 2.0 mL/min helium as the carrier gas, and an injection volume of 1 μL. The detection was performed using a Thermo Fisher Trace 1300 ISQ mass spectrometer (Thermo Fisher Scientific Inc., Sunnyvale, CA, USA). The initial column temperature was 100 °C and then increased to 140 °C at 7.5 °C/min, followed by an increase to 250 °C at 60 °C/min, and finally held at 250 °C for 10 min (total run time of 17.17 min). The data were handled with GC/MS Postrun Analysis software.

### 2.11. Statistical Analysis

Data were statistically analyzed using Prism 7.0 (GraphPad Software Inc., San Diego, CA, USA) and expressed as mean ± standard deviation (SD). The differences among multiple comparisons were analyzed using one-way ANOVA followed by Fisher’s LSD test. Statistical significance was assessed at *p* < 0.05.

## 3. Results

### 3.1. Huangjiu Feeding Increased Body Weight, Lowered the Liver Index, Suppressed Hepatic Steatosis, and Mitigated Intestinal Damage Relative to EtOH Feeding

The effects of chronic alcohol exposure on the body weight and liver index of mice are represented in Figure 1B,C. As no significant difference was found in initial body weight among the different groups, the alcohol exposure reduced the body weight of mice significantly (Figure 1B; *p* < 0.001). The body weights of the PC and Huangjiu groups decreased remarkably compared to that of the Ctrl group. Notably, they were distinctly higher than the body weight of the EtOH group, except that no significant difference was detected between Huangjiu B and EtOH groups. Compared with the Ctrl group, the liver index (liver weight: body weight ratio) of the other five groups significantly increased (Figure 1C; *p* < 0.001). However, the liver index of mice in the PC and Huangjiu groups was strikingly lower than that in the EtOH group, demonstrating that the NAC in Huangjiu might alleviate EtOH-induced liver swelling. In addition, there was no significant difference in the body weight and the liver index among the three Huangjiu groups. Pathological changes in the murine liver and ileum in each group are shown in Figure 1J–L. In the Ctrl group, the structure of the hepatic lobule was normal, the liver plates were neatly arranged, and the morphology of hepatocytes was normal (Figure 1J). Severe steatosis and lipid droplet accumulation was seen in the mouse liver in the EtOH group (Figure 1J,K). Additionally, the arrangement of hepatocytes was disordered, the cytoplasm of hepatocytes around the central vein was swollen and deformed, and inflammatory cells occasionally infiltrated in spots. PC intervention effectively alleviated these pathological conditions, and only a small amount of microbubble steatosis of hepatocytes was detected. Figure 1J,K illustrate that the number of fat vacuoles and lipid droplets in hepatocytes of mice in three Huangjiu groups was significantly less than that in the EtOH group. The cytoplasm of hepatocytes contained only tiny circular vacuoles, and the hepatocytes around the central vein appeared slightly swollen (Figure 1J). Besides, no apparent inflammatory cell infiltration was found. In the EtOH group, as depicted in Figure 1L, the lamina propria of the ileum was slightly edemic, the villous epithelium and lamina propria of the small intestine were slightly separated, and the gap between glands became larger. The lymphocytes in the lamina propria were slightly increased. The histological morphology of the ileum in the PC and Huangjiu groups was similar to that in the Ctrl group, showing intact mucosal epithelium, abundant glands in lamina propria, and no apparent inflammatory cell infiltration. According to the above results, the degree of liver injury and ileal damage induced by Huangjiu with the same alcohol dose was remarkably lower than the EtOH group. Therefore, it was speculated that the various NAC in Huangjiu might exert hepatoprotective and intestinal-barrier-protecting activities through a synergistic effect.

### 3.2. Huangjiu Interventions Improved the Hepatic Function Indexes of Mice Relative to EtOH Intervention

As the chemical markers of liver injury, serum ALT and AST activities were detected to compare the hepatotoxicity induced by Huangjiu and pure EtOH exposure (Figure 1D,E). Compared to the Ctrl group, serum ALT and AST levels in the EtOH and Huangjiu groups were significantly increased (*p* < 0.001), suggesting that the liver injury occurred due to chronic alcohol consumption. In contrast, the serum transaminase activity of Huangjiu-treated mice was markedly lower than that of EtOH-treated mice. Particularly, the transaminase activity in the Huangjiu C group was not significantly different from that in the PC group (Figure 1D,E) and the serum ALT index of Huangjiu C was close to the normal level (Figure 1E). Besides, no significant difference in the restoration of serum ALT and AST levels was observed in mice between Huangjiu A and B groups (Figure 1D,E).

The degree of liver damage caused by alcohol could also be characterized quantitatively by measuring the TC and TG contents in the serum (Figure 1F,G) and liver (Figure 1H,I) of mice. Compared with the Ctrl group, the serum and liver profiles of TC and TG levels in EtOH and Huangjiu groups were significantly increased (*p* < 0.001), indicating the appearance of the fat accumulation. In comparison to mice receiving pure EtOH, Huangjiu interventions remarkably suppressed the increases in TC and TG levels in mice (*p* < 0.001 in Figure 1F–I). In addition, the recovery effects of Huangjiu A and C on the above hepatic biomarkers were better than that of Huangjiu B (Figure 1F–I). Notably, the serum TC in the PC and Huangjiu C groups was close to the Ctrl level (Figure 1F). No statistically significant difference was found in hepatic lipid levels between the three Huangjiu groups (Figure 1I). The quantitative determination findings of TC and TG in mice were consistent with the findings of the pathological liver analysis (Figure 1J,K).

### 3.3. The Antioxidant Defense System of Huangjiu-Treated Mice Was More Complete Than That of EtOH-Treated Mice

**Figure 2:**
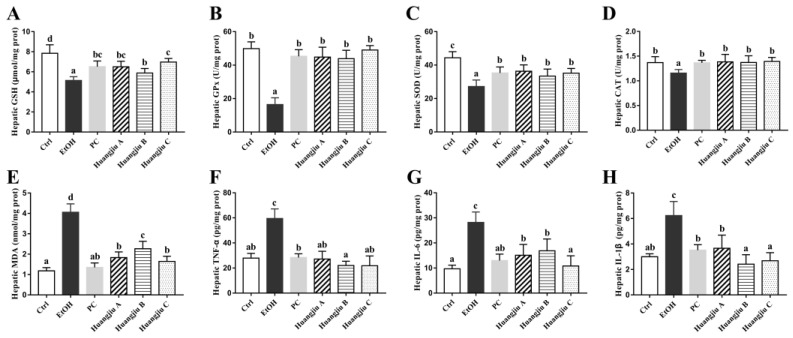
Effects of Huangjiu interventions on hepatic oxidative stress parameters (**A**–**E**) and inflammatory indicators in mice (**F**–**H**). (**A**) Hepatic GSH level. (**B**) Hepatic GPx activity. (**C**) Hepatic SOD activity. (**D**) Hepatic CAT activity. (**E**) Hepatic MDA level. (**F**) Hepatic TNF-α level. (**G**) Hepatic IL-6 level. (**H**) Hepatic IL-1β level. Bar plot shown as mean ± standard deviation (SD) (*n* = 10). Different lowercase letters indicate statistically significant differences (*p* < 0.05) between the groups using one-way ANOVA followed by Fisher’s LSD test. GSH, glutathione; GPx, glutathione peroxidase; SOD, superoxide dismutase; CAT, catalase; MDA, malonyldialdehyde; TNF-α, tumor necrosis factor-α; IL-6, interleukin-6; IL-1β, interleukin-1β.

Extensive alcohol consumption might lead to an imbalance in the antioxidant defense system, therefore, inducing hepatic oxidative stress. The effects of Huangjiu interventions on endogenous non-enzymatic (GSH) and enzymatic antioxidant (GPx, SOD, and CAT) systems in mice are represented in Figure 2A–D. Chronic pure EtOH exposure evidently reduced the GSH level and dramatically decreased the activities of antioxidant enzymes, including SOD and CAT, in the liver of mice when compared to the Ctrl group (*p* < 0.001 in Figure 2A–C and *p* < 0.01 in Figure 2D), indicating the presence of hepatic oxidative damage. The alcohol-induced GSH depletion and the declines in GPx, SOD, and CAT activities were, however, remarkably ameliorated in Huangjiu groups, with the same trend as the PC administration. Notably, the activities of hepatic GPx and CAT in the three Huangjiu groups were comparable to those in the Ctrl group (Figure 2B,D). Besides, no statistically significant difference was found in hepatic levels of GPx, SOD, and CAT between PC and the three Huangjiu groups (Figure 2B–D). As a product of lipid peroxidation, MDA may reflect the degree of lipid peroxidation and the severity of hepatocyte injury caused by the free radical attack. As expected, the hepatic MDA concentration in EtOH-fed mice was the highest, followed by Huangjiu-fed mice (Figure 2E). Compared with pure EtOH intake, Huangjiu intake conspicuously ameliorated the hepatic oxidative stress parameters (*p* < 0.001), accounting for less hepatotoxicity observed in Huangjiu groups (Figure 1J,K).

### 3.4. Huangjiu Feeding Resulted in a Lower Level of Hepatic Inflammatory Factors in Mice Than EtOH Feeding

As protein mediators of inflammation, pro-inflammatory cytokines are involved in hepatic steatosis, inflammatory response, and apoptosis [35,36]. The hepatic levels of inflammatory factors TNF-α, IL-6, and IL-1β were determined to evaluate the influences of Huangjiu and EtOH administrations on inflammatory response in the liver of mice (Figure 2F–H). The levels of TNF-α, IL-6, and IL-1β in the EtOH group were remarkably higher than that in the Ctrl group (*p* < 0.001), indicating the occurrence of liver inflammation. Compared to the EtOH group, Huangjiu interventions significantly suppressed the release of cytokines (*p* < 0.001), which might partially relieve liver inflammation and injury. Notably, the TNF-α and IL-1β levels of Huangjiu groups were not remarkably different from those of the Ctrl group (Figure 2F,H), demonstrating the potential of NAC in Huangjiu to diminish liver inflammation. Additionally, the NAC in Huangjiu C group showed the highest potential for reducing liver inflammation among the three Huangjiu groups.

### 3.5. Huangjiu Interventions Improved the Integrity of Intestinal Barrier in Mice Relative to EtOH Intervention

**Figure 3:**
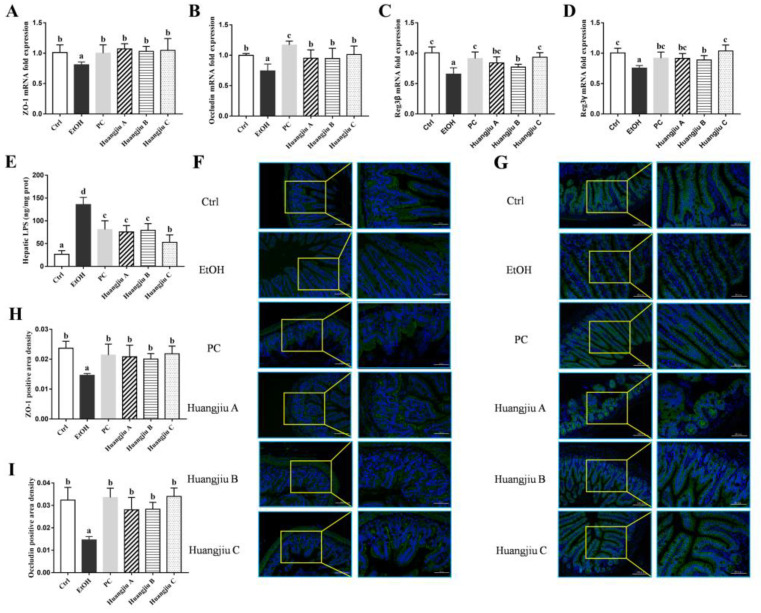
Effects of Huangjiu interventions on intestinal barrier functions in mice. The mRNA expression levels of ileum ZO-1 (**A**), occludin (**B**), Reg3β (**C**)and Reg3γ (**D**) were determined by RT-qPCR using GAPDH as an internal control. (**E**) Levels of hepatic LPS. Representative images and quantified fluorescence intensity of immunofluorescence analysis of ZO-1 (**F**,**H**) (green) and occludin (**G**,**I**) (green) in ileum of mice. Nuclei were stained with DAPI (blue). Magnification ×200 and ×400. Bar plot shown as mean ± standard deviation (SD) (*n* = 10). Different lowercase letters indicate statistically significant differences (*p* < 0.05) between the groups using one-way ANOVA followed by Fisher’s LSD test. LPS, lipopolysaccharides.

The mRNA levels of TJ proteins zonula occludens-1 (ZO-1) and occludin in mice ileum were analyzed by RT-qPCR, and the results are shown in Figure 3A,B. Compared to the Ctrl group, the mRNA levels of ZO-1 and occludin remarkably decreased following pure EtOH exposure (*p* < 0.05 in Figure 3A and *p* < 0.01 in Figure 3B), demonstrating that chronic EtOH feeding led to the destruction of TJ and intestinal barrier function in mice. However, Huangjiu administrations markedly increased the mRNA levels of TJ proteins compared with EtOH administration. Meanwhile, immunofluorescence was used to determine the expression of intestinal-barrier-related functional proteins (Figure 3F–I). Although the fluorescence intensity of ZO-1 and occludin in Huangjiu groups was slightly lower than that in the Ctrl group, the expression levels of ZO-1 and occludin in Huangjiu groups were significantly higher than those in the EtOH group. Besides, no statistically significant difference was observed in both the mRNA (Figure 3A,B) and protein (Figure 3F–I) expression levels of ZO-1 and occludin among the three Huangjiu groups. The findings demonstrated that Huangjiu-fed mice had a more complete intestinal barrier function than EtOH-fed mice, which was consistent with the results of ileum histopathological analysis (Figure 1L).

To further investigate the impacts of Huangjiu and EtOH intake on intestinal homeostasis, the expression levels of antimicrobial peptides regenerating islet-derived protein (Reg3β and Reg3γ) in the ileum were observed. The mRNA levels of Reg3β and Reg3γ in EtOH-treated mice were significantly decreased (*p* < 0.001 in Figure 3C,D). However, Huangjiu feeding conspicuously up-regulated the mRNA expression levels of Reg3β and Reg3γ compared with EtOH feeding. Notably, the mRNA expression levels of Reg3β and Reg3γ in Huangjiu A and C groups exhibited no significant difference from that in the PC group (Figure 3C,D). These results suggested that Huangjiu interventions elevated the mRNA expression levels of TJ proteins and antimicrobial peptides, thereby enhancing intestinal integrity, barrier function, and intestinal homeostasis in mice compared with pure EtOH intervention.

Maintaining the integrity of intestinal barrier function might prevent intestinal microbes and their metabolites from entering portal vein circulation through the intestinal barrier. The content of hepatic LPS was analyzed, and the results are presented in Figure 3E. The LPS level was elevated in Huangjiu-treated mice in comparison to the Ctrl mice but remarkably lower than that in EtOH-treated mice (*p* < 0.001), implying that the effective interventions of Huangjiu NAC in ALD might be attributed to a reduction in intestinal permeability, as well as a decrease in translocated LPS-induced endotoxemia. Additionally, the LPS content in Huangjiu A and B groups was not significantly different from that of the PC group. The Huangjiu C group presented significantly lower LPS levels than the PC group (Figure 3E).

### 3.6. Huangjiu and EtOH Treatments Resulted in Divergent Intestinal Community Structures and Gut Microbiota Profiles

**Figure 4:**
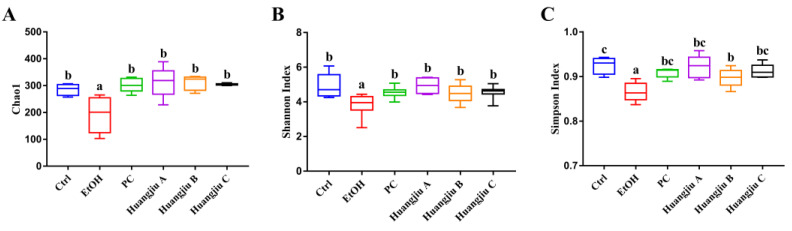

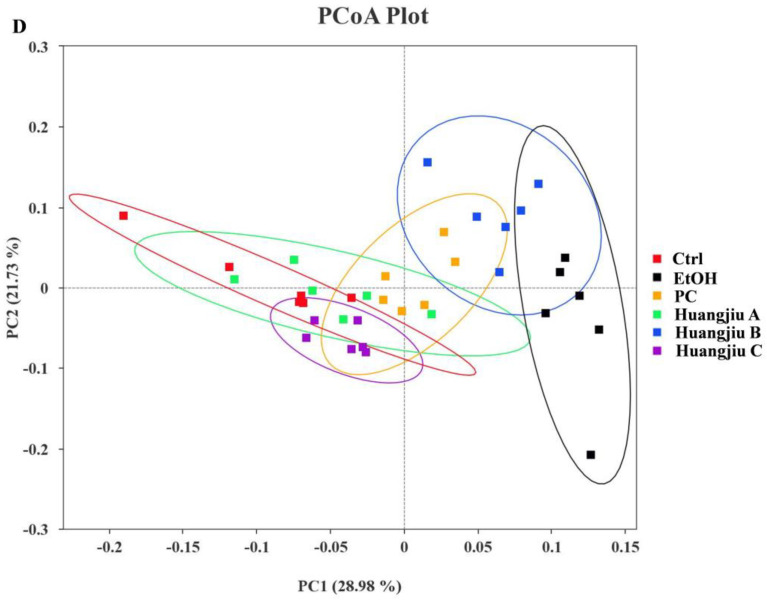
Effects of Huangjiu interventions on α-diversity and β-diversity of gut microbiota. (**A**) Chao1 richness index of gut microbiota. (**B**) Shannon diversity index of gut microbiota. (**C**) Simpson diversity index of gut microbiota. (**D**) Weighted Unifrac PCoA plot of gut microbial communities based on the OTU data. All data expressed as mean ± standard deviation (SD) (*n* = 8). Different lowercase letters indicate statistically significant differences (*p* < 0.05) between the groups using one-way ANOVA followed by Fisher’s LSD test.

The results of the α-diversity analysis are shown in Figure 4. The richness index (Chao1) and diversity indexes (Shannon index and Simpson index) of the gut microbiota were remarkedly decreased in the EtOH group compared to the Ctrl group (*p* < 0.01 in Figure 4A,B and *p* < 0.001 in Figure 4C), while these deteriorating changes were reversed in Huangjiu groups (Figure 4A–C). In addition, the α-diversity of Huangjiu groups was not significantly different from that of the PC group. According to the β-diversity analysis of the weight UniFrac PCoA algorithm, a clear separation was seen between the EtOH group and the Ctrl group, indicating that the gut microbiota structure of mice was dramatically shifted by pure EtOH feeding (Figure 4D). Furthermore, the PC, Huangjiu A, and Huangjiu C groups were distinguished from the EtOH group. Huangjiu A, Huangjiu C, and Ctrl groups were closer than Huangjiu A, Huangjiu C, and EtOH groups, suggesting that the microbial community structure of Huangjiu A and Huangjiu C groups was related more closely to that of the Ctrl group than that of the EtOH group. These data demonstrated that interventions in Huangjiu NAC could regulate the gut microbiota structure disrupted by EtOH.

**Figure 5:**
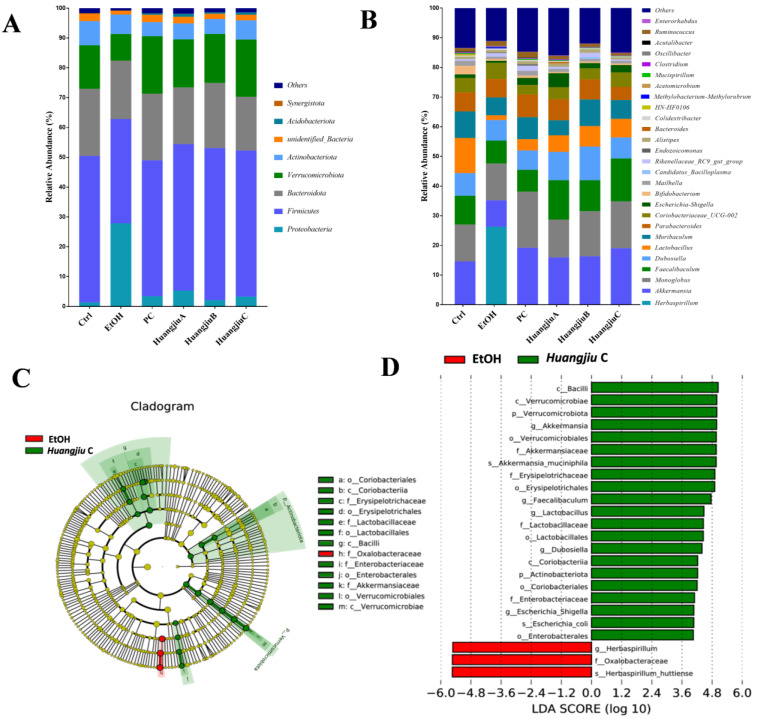
Effects of Huangjiu interventions on gut microbiota profiles. (**A**) Gut microbiota composition at the phylum level. (**B**) Gut microbiota composition at the genus level. (**C**,**D**) LEfSe comparison of gut microbiota between EtOH and Huangjiu C groups.

**Figure 6:**
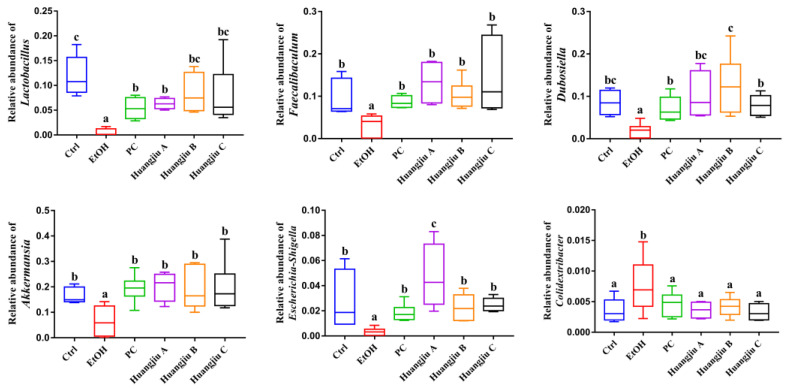
The relative abundance of gut microbial community members at the genus level. Values presented as mean ± standard deviation (SD) (*n* = 8). Different lowercase letters indicate statistically significant differences (*p* < 0.05) between the groups using one-way ANOVA followed by Fisher’s LSD test.

This study also attempted to analyze fecal bacterial community profiles at different taxonomic levels to give insights into specific differences in the gut microbiome resulting from the interventions of Huangjiu and EtOH (Figure 5). At the phylum level, compared with the Ctrl group, the relative abundance of *Firmicutes*, *Bacteroidetes*, *Verrucomicrobia*, and *Acidobacteriota* in the EtOH group decreased, whereas the relative abundance of *Proteobacteria* increased (Figure 5A), indicating that pure alcohol exposure led to the disturbance of gut microbiota in mice. However, in Huangjiu groups, these alterations were restored to similar levels to those in the Ctrl group. At the genus level, the relative abundance of *Lactobacillus*, *Faecalibaculum*, *Dubosiella*, *Akkermansia*, and *Escherichia-Shigella* significantly decreased, whereas the relative abundance of *Herbaspirillum* and *Colidextribacter* dramatically increased after pure alcohol exposure, while these trends were significantly reversed in Huangjiu groups (Figure 5B and Figure 6). Subsequently, LEfSe analysis was used to identify the most differentially specific bacterial taxa that were predominant in EtOH and Huangjiu C groups (Figure 5C,D). The findings revealed that the gut microbiota of the Huangjiu C group was enriched by *Akkermansia*, *Faecalibaculum*, *Lactobacillus*, *Dubosiella*, and *Escherichia-Shigella*, which was in discordance with that of the EtOH group. Conversely, the *Herbaspirillum* was more dominant in the EtOH group than in the Huangjiu C group. These findings indicated that Huangjiu NAC administrations could restore the alterations in intestinal community structures and attenuate gut microbiota dysbiosis caused by EtOH.

### 3.7. Huangjiu and EtOH Intake Resulted in Distinct SCFA Levels

**Figure 7:**
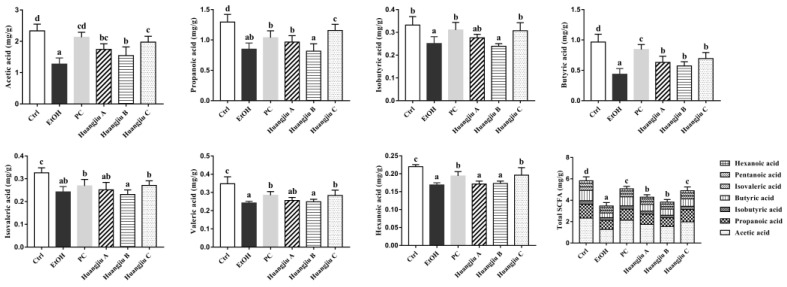
Effects of Huangjiu interventions on the levels of fecal short-chain fatty acids (SCFAs). Data expressed as mean ± standard deviation (SD) (*n* = 10). Different lowercase letters indicate statistically significant differences (*p* < 0.05) between the groups using one-way ANOVA followed by Fisher’s LSD test.

GC-MS quantitatively analyzed seven kinds of SCFAs in fecal samples to understand the alterations in intestinal-microbiota-metabolized SCFAs caused by chronic alcohol feeding (Figure 7), including acetic acid, propionic acid, isobutyric acid, butyric acid, isovaleric acid, valeric acid, and hexanoic acid. The results showed that the levels of seven SCFAs in the EtOH group were dramatically decreased compared to the Ctrl group (*p* < 0.001), suggesting that pure alcohol intake could significantly reduce intestinal SCFA content. The levels of SCFAs, except for isovaleric acid, in feces in the Huangjiu C group were especially higher than those in the EtOH group. Additionally, no statistically significant difference was found in the SCFA levels between the PC and the Huangjiu C groups, except for propionic acid and butyric acid. The Huangjiu A and Huangjiu B groups showed markedly increased levels of acetic acid and butyric acid than the EtOH group, whereas the levels of the other five SCFAs exhibited no significant difference. Furthermore, it was found that Huangjiu interventions exhibited higher total SCFAs content in the intestine than the EtOH intervention. These results revealed that the NAC in Huangjiu might alleviate EtOH-induced liver injury by promoting the production of intestinal SCFAs.

## 4. Discussion

Alcohol consumption is attributed to its unique social and cultural characteristics around the world. Alcohol abuse, however, has created an enormous healthcare burden and has become a global public health issue. Alcohol is one of the most frequent inducers of liver disease, among which ALD is a common chronic disease that threatens human health. Several studies have revealed that various bioactive components in fermented foods exhibit hepatoprotective activities and have the advantages of low toxic side effects, and being multi-pathway and multi-target [37,38,39,40]. The increasing demand by consumers for nutritious and healthy food has led to an increased focus on improving the functionality of NAC in fermented alcoholic beverages to alleviate the health injuries caused by alcohol. Huangjiu, a traditional fermented food with multi-strain mixed fermentation, is recognized as one of the three ancient wines globally, together with beer and grape wine. The abundant nutrients and bioactive compounds were produced during the long-term brewing process under the unique bilateral fermentation and micro-ecosystem of Huangjiu [25,28,41,42]. Functional analysis of Huangjiu NAC evaluated their beneficial effects on antioxidant, immunomodulatory, anti-tumor, atherosclerosis prevention, gut microbiota restoration, and intestinal micro-ecology improvement [25,28,30,32,33]. We, therefore, hypothesized that these bioactive compounds in Huangjiu might also inhibit the progression of ALD. This study aimed to compare the divergent hepatotoxicity between Huangjiu and pure EtOH under the premise of the same alcohol dosage to evaluate the possible hepatoprotective effects of NAC in Huangjiu.

According to the literature, chronic alcohol consumption disrupts the normal metabolism of hepatic lipids by increasing fatty acid synthesis and reducing the oxidation and transport of fatty acids [43]. Based on the representative pathological images of liver samples, it was found that substantial hepatocellular steatosis and severe accumulation of fat droplets occurred in the EtOH group. Interestingly, the number of fat vacuoles and lipid droplets in Huangjiu groups was remarkably less than that in the EtOH group, suggesting a lesser pathological injury in Huangjiu-fed mice. Moreover, the quantitative determination results of TC and TG in serum and liver were consistent with the histological analysis. A previous study demonstrated that Huangjiu peptides could efficiently alleviate aberrant hepatic lipid metabolism and regulate the serum and hepatic biomarker levels in hyperlipidemic mice [32]. Therefore, it was hypothesized that the NAC in Huangjiu might contribute to the attenuation of abnormal serum lipid metabolism and liver lipid accumulation induced by alcohol. Serum AST and ALT are biomarkers that reflect liver function. In line with other studies, the serum transaminase levels in the EtOH group were remarkably elevated after chronic alcohol feeding [13,39]. Notably, the NAC in Huangjiu could strikingly antagonize the elevation of ALT and AST levels caused by alcohol consumption and alleviate liver dysfunction.

Oxidative stress is a state of imbalance between pro-oxidants and antioxidants, which plays a vital role in the pathogenesis of ALD. The EtOH-induced surge of ROS and decreased antioxidant enzyme activities in the liver could lead to hepatic oxidative stress and impair hepatocytes. MDA is produced through the lipid decomposition of peroxides. It is generally regarded as a critical indicator of lipid peroxidation in vivo [44]. This study measured antioxidant parameters, including GSH, GPx, SOD, CAT, and MDA, in the murine liver to evaluate the hepatic oxidative stress. As expected, Huangjiu interventions remarkably reversed the GSH depletion, MDA elevation, and the decreasing of GPx, SOD, and CAT activities relative to EtOH intervention. Additionally, the lipid peroxidation products could mediate the inflammatory response in the liver. Moreover, the adducts of acetaldehyde, ROS, and protein neoantigens could also aggravate the inflammation [45]. It was signified that the TNF-α, IL-6, and IL-1β levels in pure EtOH-treated mice were substantially higher than those in Ctrl mice, indicating that alcohol exposure stimulated inflammatory markers generation. In agreement with the above results, slight focal inflammatory cell infiltration in the hepatocyte interstitium was observed in the H&E-stained sections of pure EtOH-treated mice. Contrary to the EtOH group, Huangjiu interventions significantly inhibited the release of inflammatory cytokines. Similar hepatoprotective effects regarding dietary components have been reported in previous studies [35,36]. The water extract of jujube contains phenols, flavonoids, and polysaccharides and is reported to prevent ALD due to its anti-inflammatory and antioxidant properties [36]. Moreover, the hydrolyzed peptide of coix seed protein with molecular weight < 5 kDa could retard lipid peroxidation via decreasing the MDA level and increasing the SOD activity in the liver, and might suppress the overexpression of serum TNF-α and IL-β-induced alcohol injury [35]. Analogously, abundant ingredients, including peptides, polyphenols, and polysaccharides, were found in Huangjiu (Table 1), whether derived from raw materials or produced by microbial metabolism. Therefore, it was hypothesized that the bioactive components in Huangjiu might be responsible for its lower hepatotoxicity compared with pure EtOH; meanwhile, the protective effects against ALD could be associated with the enhancement of antioxidant defense systems and attenuation of inflammatory response.

LPS can be transferred from the intestine to the liver via the portal vein, which induces the activation of inflammatory cells and produces pro-inflammatory cytokines [46]. Thus, the level of enterogenic endotoxin LPS in the liver was measured. The results revealed that pure EtOH feeding remarkably increased liver LPS levels in mice, according to previous studies [6,13]. Intriguingly, the hepatic LPS level in Huangjiu groups was remarkably lower than that in the EtOH group, suggesting that the bioactive components in Huangjiu were capable of inhibiting the formation of pro-inflammatory cytokines by reducing the level of hepatic LPS. It is well established that the loss of intestinal barrier integrity would result in intestinal endotoxin translocation [9]. The protective layer defensin, TJ protein, and intestinal immune cells ensure the integrity of the intestinal barrier by performing their respective functions [47]. TJs are assembled by various specific proteins. Therein, occludin could regulate the localization and function of TJ complexes via phosphorylation and can affect the permeability of TJs. Meanwhile, it can also accommodate cell adhesion and maintain the polarity of intestinal mucosal epithelial cells. ZO-1 is a scaffold protein that plays a vital role in adjusting the TJ function in the intestinal epithelium [48]. Acetaldehyde, a metabolite of EtOH, has been verified to induce tyrosine phosphorylation of ZO-1 and threonine dephosphorylation of occludin, leading to the destruction of intestinal TJ and intestinal barrier function [49,50]. In addition, antimicrobial peptides produced by intestinal epithelial cells and Paneth cells are a crucial part of the intestinal mucosal innate immune barrier [39]. They are essential for the maintenance of intestinal homeostasis and protection of the host, accompanied by intestinal symbiotic bacteria. Reg3β and Reg3γ are both secreted C-type lectins that possess bactericidal activity [51]. In this study, the mRNA levels of ZO-1, occludin, Reg3β, and Reg3γ in the ileum of Huangjiu-treated mice were significantly higher than those of pure EtOH-treated mice, implying that the functional components of Huangjiu could partially restore alcohol-induced intestinal barrier dysfunction, thus, inhibiting the exudation of enteric LPS. In the meantime, the histological study of murine ileum might reveal the crucial role of Huangjiu bioactive components in alleviating alcohol-induced intestinal injury and protecting intestinal barrier function. In line with our findings, emerging evidence demonstrated that the vinegar extracts rich in phenolic compounds dramatically inhibited the decreasing expression levels of TJ proteins and antimicrobial peptides caused by alcohol, therefore, reducing intestinal permeability and the transport of pro-inflammatory LPS [39]. Furthermore, it was stated that supplementing litchi pulp phenolic extract with procyanidins B2, rutin, and (-)-epicatechin as major components improved the EtOH-induced intestinal barrier dysfunction, proved by elevated mRNA levels of TJ proteins, antibacterial protein, and mucus-protecting proteins in the small intestine [52]. Thus, our findings revealed that the bioactive components in Huangjiu might contribute to the alleviation of ALD by enhancing intestinal integrity and barrier function and retaining intestinal homeostasis.

Substantial evidence suggests that the dysregulation of the gut microbiome might lead to intestinal barrier dysfunction, increasing the level of circulating endotoxin [13,39,53]. Intestinal microbiota dysbiosis is a prominent feature of alcohol abuse and proved to be closely associated with ALD occurrence and progression [6]. Subsequently, 16S rRNA sequencing was used to evaluate the effect of Huangjiu interventions on the composition of gut microbiota in mice. The findings of α-diversity presented that pure alcohol feeding significantly reduced the richness index and diversity indexes of gut microbiota in mice, which was consistent with previous studies [8,54]. Huangjiu groups, however, possessed substantially higher α-diversity than that of the EtOH group, indicating that the NAC in Huangjiu strikingly reversed this trend. The analysis of β-diversity (weighted UniFrac PCoA analyses) revealed that the gut microbiota of Huangjiu groups was apparently separated from that of the EtOH group but close to that of the Ctrl group, demonstrating that the bioactive components in Huangjiu partially reversed the EtOH-induced changes in gut microbiota composition. Additionally, the alterations in gut microbiota profiles were analyzed at the phylum level. According to the results, pure alcohol feeding pronouncedly reduced the relative abundance of *Firmicutes*, *Bacteroidetes*, *Verrucomicrobia*, and *Acidobacteriota*; meanwhile, it evidently increased the relative abundance of *Proteobacteria*, which was in line with previous reports [39,53]. It has been stated that the increase in the relative abundance of *Proteobacteria* would promote the growth of more pathogenic bacteria, leading to gut microbiota disorder, intestinal mucosal barrier damage, and an increase in intestinal permeability, which, subsequently, result in endotoxemia, inflammation, and related diseases [55]. However, the above changes in phylum levels were reversed in Huangjiu groups, signifying that the NAC in Huangjiu could reduce the EtOH-induced gut microbiota imbalance in mice. Based on the genus-level analysis, the relative abundance of the genera that were notably reduced in the EtOH group, such as *Lactobacillus*, *Faecalibaculum*, and *Akkermansia*, were restored in the Huangjiu groups. Symbiotic probiotic *Lactobacillus* was reported to inhibit pathogens, stimulate mucin secretion, and control inflammation, and their relative abundance could reflect the health status of the organism [13]. *Faecalibaculum* was considered an essential component of carbohydrate and energy metabolism pathways in the host [56]. In addition, *Akkermansia* colonizes the mucus layer of the human intestine, and could degrade mucin and maintain the integrity of the intestinal barrier [57]. Thus, the bioactive components in Huangjiu could prevent the disruption in intestinal barrier function by inhibiting the alcohol-induced decrease in *Akkermansia* abundance. Moreover, an evident rise in the relative abundance of *Herbaspirillum* and *Colidextribacter* was detected in the EtOH group compared with the Ctrl group. The Gram-negative bacteria *Herbaspirillum*, has a cell wall constituent (LPS) that might take part in the pathogenesis of hepatic inflammation via the gut–liver axis. Recently, *Herbaspirillum* was reported to induce bacteremia and sepsis in immunocompromised patients [58]. It was found that *Colidextribacter* was positively correlated with serum MDA and negatively correlated with serum T-AOC, SOD, and GPx levels [59]. It was noteworthy that Huangjiu feeding reversed the alterations in the aforementioned genera in comparison with pure EtOH feeding. Two bioactive peptides isolated from Huangjiu with Tyr-Val-Lys-Val and Leu-Phe-Trp sequences were found to restore the dysbiosis in the gut microbial community caused by a high-fat diet (HFD) by modulating the specific bacterial taxa [32]. According to Kiyono et al. (2013), Pyroglutamyl Leucine found in sake possessed hepatoprotective activities; meanwhile, it was confirmed to alleviate dextran sulfate sodium-induced colitis by normalizing the proportions of *Bacteroidetes* and *Firmicutes* in the colon [38]. Additionally, it was stated that red wine polyphenols could promote health by recovering disordered gut microbiota in rats, which was in harmony with our findings [60]. Collectively, the gut microbiome could be a promising target for ALD treatment. Therefore, it was hypothesized that the functional components in Huangjiu might antagonize the progression of ALD by regulating the intestinal microbiota.

Alterations in the gut microbiota would result in changes in their metabolites [61]. SCFAs are the key end products of indigestible carbohydrates fermented by intestinal microbes. An evolving body of evidence supports the crucial role of SCFA in host health [13,14,53]. The data showed that pure EtOH intervention markedly reduced the levels of seven kinds of SCFA. Compared with the EtOH group, acetic acid and butyric acid levels in Huangjiu A and B groups were remarkably increased. Besides, the levels of six kinds of SCFA in the Huangjiu C group were strikingly higher than those in the EtOH group, except for isovaleric acid. Acetic acid is the end product of EtOH metabolism [62]. A higher level of acetic acid in the feces of Huangjiu-treated mice indicated a higher detoxification effect on EtOH and acetaldehyde. Propionic acid could improve hepatic lipid metabolism and insulin sensitivity, reducing liver fat [63]. Acetic acid and propionic acid are reported to control neutrophil activation and reduce the inflammatory cascade in diabetic patients by inhibiting the expression of chemokines and cytokines [64]. Butyric acid was considered the pivotal regulator of intestinal TJ protein and was shown to enhance intestinal barrier function by increasing the expression of claudin-1 and ZO-1 [65]. It was stated that butyric acid could reduce the translocation of LPS by reversing the aberrant expression of ZO-1, thus, inhibiting macrophage activation, pro-inflammatory cytokine production, and neutrophil infiltration from alleviating liver injury in rats [66]. Furthermore, butyric acid has been demonstrated to play a potential role in immunoregulation, which could inhibit the activities of TNF-α, IL-6, and myeloperoxidase by preventing the activation of NF-kB in Kupffer cells [67]. Moreover, butyrate might exert a direct anti-inflammatory effect at the inflammation sites [68,69]. There is recent evidence that sodium butyrate administration could reduce HFD-induced chronic inflammation by modulating gut microbiota composition and intestinal barrier [70]. Supplementation with butyrate could also prevent alcohol-induced disruption of intestinal TJs and liver inflammation [71].

Huangjiu NAC interventions reversed the significant reduction in intestinal SCFAs induced by alcohol feeding, which was in line with the regulation of Huangjiu NAC on the gut microbiota of mice. There was speculation that Huangjiu interventions might increase the abundance of SCFA producers in the intestinal tract of mice compared with pure EtOH, which would lead to a higher SCFA level. Compared to the EtOH group, the higher total SCFA level of Huangjiu groups might be associated with the higher abundance of intestinal *Akkermansia*, an efficient SCFA producer, in Huangjiu-fed mice. *Akkermansia Municiphilla* has been identified as a vital species capable of producing propionic acid through mucin degradation [72]. Furthermore, the Huangjiu groups had a higher abundance of *Lactobacillus* and *Faecalibaculum* than the EtOH group. *Lactobacillus* was reported to promote intestinal health by producing lactic acid, acetic acid, and propionic acid [73]. Butyric acid producers can use acetic acid to produce butyric acid, and *Faecalibaculum* is a known butyric-acid-producing bacterium [74]. Several studies demonstrated that the decrease in intestinal bacterial diversity and the loss of butyric-acid-producing bacteria were observed in inflammatory bowel disease and metabolic abnormalities, such as type 2 diabetes mellitus, which was in accordance with our findings [75,76,77]. Collectively, the lower degree of liver injury caused by Huangjiu in relation to EtOH with the same alcohol dose might be partially due to the bioactive components in Huangjiu, which promoted the proliferation of beneficial microbes and the production of SCFAs in the intestinal tract.

In this study, three typical semi-dry Huangjiu were selected to compare the hepatotoxicity with pure EtOH at the same alcohol dose. Interestingly, all types of Huangjiu consumption elicited milder hepatic injury relative to EtOH intake. Through the comparison of phenotype and hepatic function indexes, the three types of Huangjiu exhibited similar hepatotoxicity, mainly reflected in that no significant difference was found in liver index, serum ALT, and hepatic TG among different Huangjiu groups. However, from the perspective of individual indicators, including serum AST and serum TG, NAC in Huangjiu C seems to have a better antagonistic effect on ALD. The fundamental component analysis of Huangjiu revealed that the abundant oligosaccharides and peptides <3 kDa in Huangjiu C may contribute to the attenuation of ALD progression. Though a number of studies have confirmed the biological activities of natural polysaccharides and peptides <3 kDa with liver protection [1,4,32,38], scarce information is available concerning the potential impact of oligosaccharides on alleviating alcohol-induced liver injury. Whether Huangjiu oligosaccharides could exert a protective effect against ALD by regulating the gut microbiota, intestinal barrier, and microbial metabolites is worth exploring in the future.

## 5. Conclusions

Biochemical and histopathological assessments indicated that Huangjiu had significantly less hepatotoxicity than pure EtOH under the premise of the same alcohol dosage in this chronic ALD model. Huangjiu NAC might contribute to ameliorating EtOH-induced liver injury through enhancing intestinal barrier function, restoring gut microbiota composition and promoting the production of functional SCFAs in the intestine. This study shed light on the association between dietary composition, gut microbiota diversity, metabolites (SCFAs), and function in fermented alcoholic beverages and their effects on human health. Given the complexity of the Huangjiu system, the separation and extraction of main bioactive components, the comparative study of their possible hepatoprotective activities, and the analysis of underlying metabolic regulation mechanisms are necessary to reveal their potential role in partially antagonizing EtOH-induced liver injury.

## Figures and Tables

**Table 1:** Main components in three types of Huangjiu.

Assays	Huangjiu A	Huangjiu B	Huangjiu C
Ethanol (% *v/v*)	14.24 ± 0.01 b	13.62 ± 0.07 a	14.46 ± 0.12 c
Total Protein (g/L)	11.55 ± 0.34 b	9.71 ± 0.14 a	12.16 ± 0.15 c
Total Phenolics (mg of GAE/L)	622.35 ± 3.87 b	572.73 ± 5.76 a	695.45 ± 3.94 c
Free Amino Acids (mg/L)	2834.74 ± 25.35 a	3101.98 ± 9.75 b	3174.10 ± 20.40 c
Total Sugars (g/L)	39.66 ± 0.71 b	33.95 ± 1.06 a	39.48 ± 0.71 b
Oligosaccharides (g/L)	3.63 ± 0.08 a	4.74 ± 0.16 b	11.83 ± 0.29 c
Polysaccharides (g/L)	9.07 ± 0.26 c	2.15 ± 0.04 a	3.85 ± 0.10 b
Peptides < 3 kDa (g/L)	2.22 ± 0.07 a	2.79 ± 0.05 b	5.72 ± 0.13 c

Data were mean ± standard deviation (SD) (*n* = 3). Values in the same row with different lowercase letters were significantly different (*p* < 0.05) by LSD’s test.

**Table 2:** Primer sequences used for qRT-PCR assay.

Gene	Primer Sequences (5′-3′)	Length
mus GAPDH	F 5′-CCTCGTCCCGTAGACAAAATG-3′ R 5′-TGAGGTCAATGAAGGGGTCGT-3′	133 bp
mus ZO-1	F 5′-GGGAAAACCCGAAACTGATG-3′ R 5′-GCTGTACTGTGAGGGCAACG-3′	103 bp
mus Occludin	F 5′-TCACTTTTCCTGCGGTGACT-3′ R 5′-GGGAACGTGGCCGATATAATG-3′	138 bp
mus Reg3β	F 5′-GCGCTGAGGCTTCATTCTTGT-3′ R 5′-TGTTACTCCATTCCCATCCACC-3′	129 bp
mus Reg3γ	F 5′-GTGCCTATGGCTCCTATTGCTA-3′ R 5′-ACCTCTGTTGGGTTCATAGCC-3′	221 bp

## Data Availability

The data are kept in the National Engineering Research Center of Cereal Fermentation and Food Biomanufacturing, School of Food Science and Technology, Jiangnan University.

## Supplementary Materials

The following supporting information can be downloaded at: https://www.mdpi.com/article/10.3390/foods11111537/s1, Supplementary Methods: Analysis of the main components in Huangjiu; Table S1: Regular-type Lieber-DeCarli liquid diet formula.
