# Supplementary material for: Non-Alcoholic Components in Huangjiu as Potential Factors Regulating the Intestinal Barrier and Gut Microbiota in Mouse Model of Alcoholic Liver Injury
Source: Foods. 2022 May 24;11(11):1537. doi: 10.3390/foods11111537 (PMC9180658; doi:10.3390/foods11111537)
Supplement: Supplementary file 1 [file foods-11-01537-s001.zip › foods-1655314-supplementary.pdf]

Supplementary Information

1. Supplementary Methods

Analysis of the main components in Huangjiu

Ethanol was quantified by gas-liquid chromatography (GLC) using a Trace 1300 gas chromatograph (Thermo Fisher Scientific Inc., MA, USA) equipped with a flame ionization detector [1]. The total protein content of Huangjiu was determined by Kjeldahl method using 6.25 as a conversion factor [2]. The concentration of phenolic compounds in Huangjiu was measured according to Folin-Ciocalteu colorimetry previously published by Liu et al. (2017), and results were presented as gallic acid equivalents [3]. An Agilent 1100 HPLC system coupled with a diode array detector (DAD) (Agilent Corp., Karlsruhe, Germany) using OPA/FMOC derivatives was used to determine the free amino acids content in Huangjiu referring to the method described previously [4]. Total sugars and total reducing sugars in Huangjiu were detected by phenol-sulfuric acid method and 3,5-dinitrosalicylic acid colorimetry (DNS) method, respectively, using glucose as the standard. The contents of oligosaccharides, polysaccharides and peptides were determined according to the methods reported by Cai, Chen, Ma, Yang, and Sun (2019) with some modifications [5]. Briefly, 2 L Huangjiu was measured accurately and ultrafiltered through a polyethersulfone membrane with cut-off value of 3 kDa (Shaoxing Haina Membrane Technology Co., Ltd. Shaoxing, China). The ultrapure water was continuously added to the feed tank until the permeate volume achieved 100 L. Thereafter, the permeate liquid was collected for the following measurements. The content of Huangjiu peptides in the $<$ 3 kDa fraction was measured by Bradford protein concentration assay kit (Beyotime Biotechnology, Shanghai, China) using bovine serum albumin as a reference substance. The contents of oligosaccharides and polysaccharides in Huangjiu were calculated according to the following formulas:

$$\text{Total oligosaccharides content} = (\text{Total sugars content in permeate liquid}) - (\text{Total reducing sugars content in permeate liquid}) \quad (1)$$

$$\text{Total polysaccharides content} = (\text{Total sugars content in Huangjiu}) - (\text{Total reducing sugars content in Huangjiu}) - (\text{Total oligosaccharides content in Huangjiu}) \quad (2)$$

All analyses were conducted in at least triplicates.

2. Supplementary Table

Table S1. Regular-type Lieber-DeCarli liquid diet formula

Ingredients	Unit calorific value	Weight (gram)	Calories(kcal)
Caisein	4.00	44.00	176.00
L-Cystine	4.00	0.50	2.00
DL-Methionine	4.00	0.30	1.00
Dextrin-maltose	4.00	115.03[a]	460.10
Cellulose	0	10.00	0
Xanthan Gum	0	3.00	0
Corn Oil	9.00	8.33	75.00
Oliver Oil	9.00	27.89	251.00
Safflower Oil	9.00	2.67	24.00
Mineral Mix	0.47	4.04	1.90
Vitamin Mix	3.80	2.50	9.00
Choline Bitartrate	0	0.53	0
Distilled Water	0	781.21	0

[a]In ethanol liquid diet formula, replaced by 45.03 gram of dextrin-maltose and 40 gram of ethanol.

References

[1] Canonico, L.; Comitini, F.; Oro, L.; Ciani, M. Sequential Fermentation with Selected Immobilized Non-Saccharomyces Yeast for Reduction of Ethanol Content in Wine. *Front Microbiol*. **2016**, *7*, 278. https://doi.org/10.3389/fmicb.2016.00278.

[2] Bravo, F. I.; Mas-Capdevila, A.; López-Fernández-Sobrino, R.; Torres-Fuentes, C.; Mulero, M.; Alcaide-Hidalgo, J. M.; Muguerza, B. Identification of novel antihypertensive peptides from wine lees hydrolysate. *Food Chem*. **2022**, *366,* 130690. https://doi.org/https://doi.org/10.1016/j.foodchem.2021.130690.

[3] Liu, N.; Yang, M.; Huang, W.; Wang, Y.; Yang, M.; Wang, Y.; Zhao, Z. Composition, antioxidant activities and hepatoprotective effects of the water extract of Ziziphus jujuba cv. Jinsixiaozao. *RSC Adv.* **2017**, *7(11),* 6511-6522. http://dx.doi.org/10.1039/c6ra27516h.

[4] Gong, M.; Zhou, Z. L.; Yu, Y. J.; Liu, S. P.; Zhu, S. H.; Jian, D. Z.; Mao, J. Investigation of the 5-hydroxymethylfurfural and furfural content of Chinese traditional fermented vinegars from different regions and its correlation with the saccharide and amino acid content. *LWT-FOOD SCI TECHNOL. **2020**, 124*. https://doi.org/10.1016/j.lwt.2020.109175.

[5] Cai, M.; Chen, S.; Ma, Q.; Yang, K.; Sun, P. Isolation of crude oligosaccharides from Hericium erinaceus by integrated membrane technology and its proliferative activity. *Food Hydrocolloids*. **2019**, *95,* 426-431. https://doi.org/https://doi.org/10.1016/j.foodhyd.2019.04.068.
